# Development of a theory-based intervention to increase cognitively able frail elders’ engagement with advance care planning using the behaviour change wheel

**DOI:** 10.1186/s12913-021-06548-4

**Published:** 2021-07-20

**Authors:** S. Combes, G. Forbes, K. Gillett, C. Norton, C. J. Nicholson

**Affiliations:** 1 Florence Nightingale Faculty of Nursing, Midwifery and Palliative Care, King’s College London, James Clerk Maxwell Building, 57 Waterloo Road, London, SE1 8WA UK; 2 St Christopher’s Hospice, London, UK; 3 Centre for Behaviour Change, University College London, London, UK; 4 Faculty of Health and Medical Sciences, Surrey University, Guildford, UK

**Keywords:** Frail elderly, Advance care planning, Communication, End-of-life care, Palliative care, Intervention development, Behavioural change, Behaviour change wheel, COM-B

## Abstract

**Background:**

Advance care planning (ACP) conversations support people to think about, discuss and document their beliefs, values and preferences regarding future care. This process means that should the person loose capacity in the future, care can be provided, consistent with their personal values and beliefs. The ACP process is particularly relevant for older people living with frailty (frail elders) as they are vulnerable to sudden deterioration. However, ACP is rarely undertaken by frail elders. The aim of this study was to develop an intervention to increase multidisciplinary health and social care professionals’ (H&SCPs) engagement of cognitively able, domestic-dwelling frail elders with ACP.

**Methods:**

Intervention development was guided by the Medical Research Council framework for complex interventions and the Behaviour Change Wheel. Multiple methods were used to understand ACP barriers and enablers: a systematic integrative review, a survey (*n* = 73 H&SCPs), and semi-structured interviews (*n* = 10 frail elders, *n* = 8 family members). A conceptual model, developed from the integrative review, underpinned data collection for the survey and interviews. Synthesis of this data, including patient and public involvement, was then used to identify H&SCPs behaviours that needed to change for ACP to be implemented and decide content and implementation for the intervention.

**Results:**

Following the Behaviour Change Wheel system, and based on the findings of the review, survey and interviews, the prototype intervention, Conversations on Living and Dying (CLaD), was developed. The CLaD prototype consisted of one 3.5-hour educational skills session for H&SCPs supported by a toolkit. Content focussed on the relevance of ACP for frail elders, experience of ACP by frail elders, and strategies H&SCPs could adopt to encourage frail elders’ engagement with ACP. Strategies include recognising the importance of relationships and living well now, preparing frail elders for ACP conversations and starting ACP early. Participants who took part in initial prototype refinement reported that the intervention helped them think differently about ACP and encouraged them to engage with frail elders.

**Conclusions:**

The use of behavioural theory enabled the development of CLaD, an evidence-based, theory-driven, person-centred intervention to support ACP engagement with frail elders. While feasibility testing is required, initial prototype refinement demonstrated that H&SCPs found the intervention to be acceptable, engaging, and clinically valuable in their practice with frail elders and their families.

**Supplementary Information:**

The online version contains supplementary material available at 10.1186/s12913-021-06548-4.

## Background

One in six community-dwelling adults aged 60 and above are estimated to be frail [1]. With the global trend of population ageing [2], older people living with frailty (frail elders) are projected to become one of the largest future users of palliative care [3]. Frailty is a syndrome of ageing, characterised as a progressive, gradual decline in physical, psychological and social functions [4]. Being frail means people are more vulnerable to sudden health deteriorations, have a reduced recovery potential, and an increased risk of mortality [5–7], disability and institutionalisation [2, 8]. Frail elders have a similar overall symptom burden as patients treated within specialist palliative care services [9]. However, these specialist needs are rarely recognised, meaning frail elders often have multiple unmet needs and experience suboptimal end-of-life care [10].

Advance care planning is defined as “*a process that supports adults at any age or stage of health in understanding and sharing their personal values, life goals, and preferences regarding future medical care*” [11]. The ACP process encourages people, often in collaboration with family members and multidisciplinary health and social care professionals (H&SCPs), to think about their wishes, beliefs and values, and plan for the end of their life [12, 13]. These conversations enable people to discuss what matters most for them and to make plans that can be referred to if the individual loses capacity in the future [11, 14]. The ACP process is particularly relevant to frail elders due to their extreme vulnerability to sudden deterioration. However, their engagement with ACP is uncommon for multiple, complex reasons including lack of clarity around what ACP means, and lack of a terminal diagnosis [15–18]. This means priorities are often not discussed prior to significant deterioration [19], leading to crisis decision making which the person may not have capacity for [8, 20], a concern that has been highlighted during the COVID-19 pandemic [21].

**Fig. 1:**
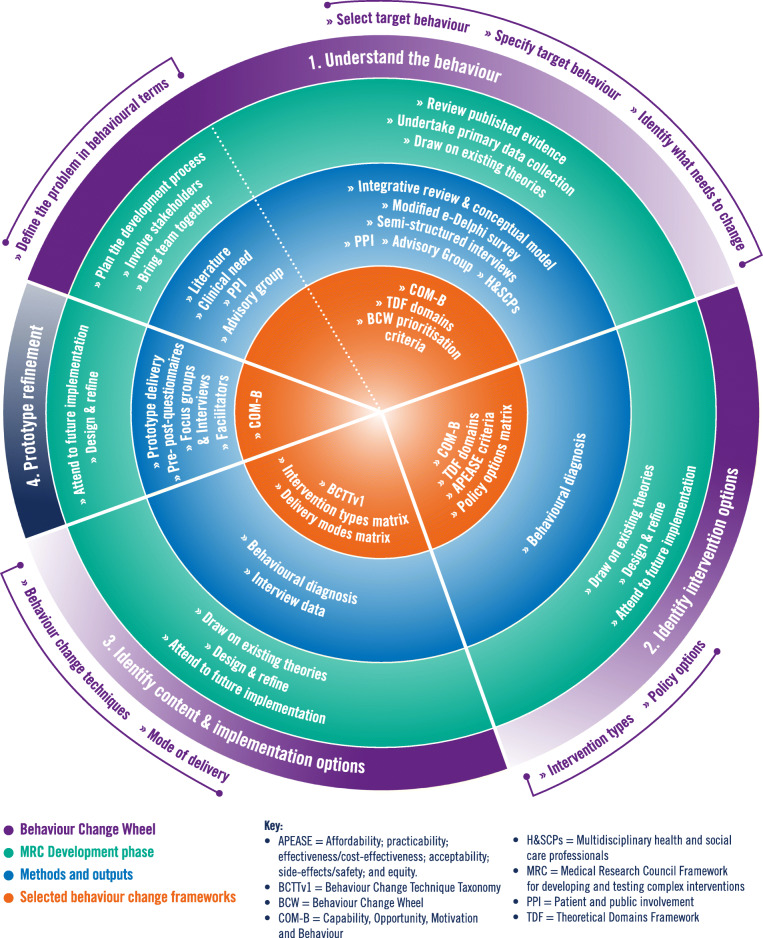
Intervention development process mapped to BCW stages and the MRC framework development phase ***PPI:*** Patient and public involvement consultation was held with individual frail elders, carers, and a frail elders and carers group, multidisciplinary health and social care professionals (H&SCPs), and informal carers in community settings. ***Advisory Group:*** This comprised PPI, clinicians, a voluntary sector representative and academics. ***MRC development phase:*** Based on O’Cathain et al. [38]

Several interventions have sought to increase ACP engagement for cognitively able frail elders living in domestic dwellings (now referred to as frail elders for brevity). Interventions have focused on reducing hospital or long-term care admission [22, 23], or completion of advance directives (the documentation of future decisions regarding ceilings of treatment) [24–29]. This trend agrees with the critique of ACP interventions in general, which argues interventions focus on aspects of ACP, such as advance directives, and that context, systems, multi-level stakeholders and working mechanisms are rarely considered [30]. Using a relevant theory, or theories, to underpin complex intervention development is believed to produce more effective interventions [31–33], and is advocated by the Medical Research Council (MRC) Framework for developing and testing complex interventions [34, 35]. However, another critique is that ACP interventions are often developed by experts, rather than being theoretically driven [36]. This study incorporates multi-level stakeholders, context, and experts, and is underpinned by behaviour change theory to support intervention development and establish theoretical working mechanisms [37, 38]. Advance care planning is complex process and includes a range of discreet behaviours that need to be conducted by multiple stakeholders (frail elders, family, and H&SCPs) within the wider context of health and social care systems. For example, H&SCPs need to talk to frail elders about ACP, frail elders need to engage in ACP, family members need to comply with frail elders wishes. Any intervention to increase frail elders’ engagement in ACP would therefore need to change stakeholder behaviour. As such, a behavioural change theory was used, specifically the Behaviour Change Wheel (BCW) guide to developing interventions [37]. The BCW is a comprehensive, pragmatic and systematic tool for designing theoretically underpinned behaviour change interventions. Developed by combining 19 behaviour change frameworks spanning multiple diverse disciplines and sectors [39, 40], the BCW has been used to develop multiple complex interventions, extensively in healthcare [41–46]. The BCW consists of multiple frameworks which guide the developer through the steps of the wheel to help them understand what is happening now, what needs to change, and how they can try to bring about the desired change in behaviour. These frameworks are explained in context throughout this paper and are shown as part of the intervention development process in Fig. 1.

This study followed a collaborative approach. The study was initially conceived collaboratively with key stakeholders (frail elders, family members, and H&SCPs). This included extensive patient and public involvement (PPI) with frail elders, family, informal carers and H&SCPs. Throughout the study it has benefited from the support of an Advisory Group, comprising PPI representatives, H&SCPs, voluntary sector representation, and academics. This collaborative approach focused on frail elders views, recognising frail elders as experts in their own lives [47] who have diverse experiences, knowledge and skills that can be used to identify solutions and enact positive change [48, 49]. The collaborative approach benefitted the study by ensuring the intervention kept the needs and views of all stakeholders at the forefront of the development process.

## Methods

The aim of this paper is to describe the development of an intervention to increase frail elders’ engagement with ACP. Intervention development is reported following the Guidance for the reporting of intervention development studies in health research (GUIDED) [35] (Additional file 1).

### Intervention development

The Behaviour Change Wheel (BCW) [37] was used to support intervention development. Fig. 1 presents the development process mapped to the steps of the BCW and includes the refinement of the prototype intervention. This Methods section describes the three steps of the BCW: 1. Understand the behaviour; 2. Identify which types of intervention and implementation strategies might be effective; and 3. Identify intervention content and how to deliver it. The Results section describes the intervention content and preliminary prototype refinement.

#### 1. Understand the behaviour

This intervention focuses on increasing H&SCPs engagement of frail elders in ACP discussions. However, at the outset of the study, it was not clear if the intervention would focus on frail elders, family members or H&SCPs as increasing ACP engagement potentially requires behaviour change from all these stakeholders. Therefore, to decide where to focus the intervention, initially the research team conducted a scoping review and discussed ACP for frail elders in clinical practice with PPI and Advisory group members. Multiple methods were then used to understand the barriers and enablers to frail elders’ engagement with ACP in greater detail. First a systematic integrative review was conducted. Full methods and findings are reported separately [18]. The review’s aim was to identify and synthesise the attitudes to, and necessary behaviours for, implementing ACP with community-dwelling frail elders, and to develop a theory-based conceptual model to underpin intervention development. The review’s key findings were: ACP for frail elders focuses on living well now rather than planning for death and dying; decisions are often made within relationships rather than autonomously; and ACP conversations need to start early. Additionally, the review found that education was required by all stakeholders, but was particularly relevant for H&SCPs who needed to develop further knowledge and skills in regard to engaging and facilitating ACP with frail elders.

To better understand the experiences and perspectives of stakeholders, and establish the validity of the conceptual model, we conducted a three-round survey with H&SCPs (*n* = 73), and semi-structured interviews with frail elders (*n* = 10) and family members (*n* = 8). The findings of the review were used to underpin data collection for both methods by supporting the development of statements for the survey and the topic guide for the interviews. Data collection began in February 2018 and concluded in September 2019.

Key survey findings were that H&SCPs agreed with the findings of the integrative review in all but the area of confidence in facilitating ACP. While the review found many H&SCPs lacked confidence, most survey participants (86%) reported that they were confident facilitating ACP with this population. However, survey participants were, in general, highly qualified and experienced with most (67% in survey one) being consultants, specialist practitioners, specialist registrars or advanced practitioners, and 15% clinical or service leads. Rigour in analysis was maintained by triangulating these findings with multiple sources (integrative review, interviews and being discussed with H&SCPs in clinical practice). Detailed methods and findings for the interviews are reported elsewhere [50].

Findings from the survey and interviews established that frail elders were generally receptive to ACP. Many of the barriers to frail elder’s ACP engagement were due to H&SCPs behaviours, for example, H&SCPs not engaging frail elders in ACP as they felt the conversation would be too upsetting. This led to the behavioural focus of the intervention being specified as increasing H&SCPs engagement of frail elders in ACP.

#### Setting

The modified survey was open to H&SCPs throughout the United Kingdom (UK). Participants were recruited through institutional gatekeepers, for example the British Geriatrics Society and professional networks. Interview participants, frail elders and family members, were recruited through a community-based older persons’ service run by a large UK urban hospice. Frail elder participants were purposively sampled to represent those who had and had not formerly engaged with ACP. Inclusion criteria are shown in Table 1.

**Table 1:** Inclusion criteria

Health and social care professionals (H&SCPs)	Frail elders	Family members
• Conduct ACP for frail elders; • Able to understand English well enough to consent and participate.	• Age ≥ 65; • Clinical Frailty Score^a^ 6 or 7; • Clinically assessed as likely to be in their last years of life; • Living in a domestic dwelling; • Understands English well enough to consent/participate; • Has capacity and assessed as able to cope with the research^b^; • If bereaved, this should be more than 6 months before recruitment.	• Nominated by the frail elder or referring clinician; • Understands English well enough to consent/participate; • If bereaved, this should be more than 6 months before recruitment.

^**a**.^The Clinical Frailty Scale [51] is scored from 0 to 9. Higher scores represent greater levels of frailty. A score of 6, moderate frailty, is described as requiring help with all outside activities and keeping house. A score of 7, severe frailty, is described as completely dependent for personal care. ^**b.**^Frail elders were assessed as able to cope physically, cognitively and emotionally by their clinician using the Mental Capacity Act [52] or the researcher using the processual model of consent [53]

#### Participants

Participant characteristics are reported in Table 2. Participants completed an informed consent process either as part of completing the survey or in writing (interviews). Frail elders were given the option of verbal rather than written consent. Ethical approvals were granted by King’s College London Research Ethics Committee (Survey LRS-17/18–5103 November 2017), and North West Greater Manchester Central Research Ethics Committee (Interviews 19/NW/0148 February 2019). Study site approval was granted April 2019.

**Table 2:** Participant characteristics

**SURVEY**	**Round 1** ***n*** **= 73**		**Round 2** ***n*** **= 51**		**Round 3** ***n*** **= 46**	
	**No.**	**%**	**No.**	**%**	**No.**	**%**
Nurses	35	48%	23	45%	21	46%
Doctors	21	29%	13	25%	12	26%
Allied health professionals^a^	10	14%	10	20%	9	19%
Social workers	7	9%	5	10%	4	9%
**Percentage clinical work with frail elders** (Round 1)				Mean 73.7 (range 5–100%)		
**INTERVIEWS**	**Frail elders** ***n*** **= 10**			**Family members** ***n*** **= 8**		
	Mean age 84 (range 71–95)			Relationship to frail elder Spouse *n* = 4 Son/daughter *n* = 4		

^a^ Allied health professionals were: Dietitian (*n* = 1), Paramedic (*n* = 1), Pharmacist (*n* = 1), Physician Associate (*n* = 1), Physiotherapist (*n* = 4) Speech and language therapist (*n* = 1), Research Psychologist (*n* = 1). All AHPs completed all Rounds except one physiotherapist who did not complete Round 3

#### Analysis

Data analysis was iterative and initially completed by one author (SC). Qualitative data (interviews and qualitative survey elements) were analysed thematically [54] and managed using NVivo 12 (QSR International (UK) Ltd). Quantitative data (quantitative survey elements) were reported using descriptive statistics. The findings of the conceptual model, modified survey and interviews were then were mapped to COM-B [40]. COM-B stands for Capability, Opportunity, Motivation, and Behaviour. COM-B’s premise is that to maintain a change in behaviour, people must have the physical and psychological capability, the social and physical opportunity, and sufficient motivation [40]. COM-B is at the hub of the BCW and is key to understanding behaviours in context, and diagnosing what changes are most likely to achieve the desired behaviour [37, 39]. At this point, COM-B was used as a mapping framework to synthesise and summarise the findings to establish a greater understanding of ACP for frail elders across multiple perspectives.

This synthesis established that the main barriers and enablers to ACP were that frail elders rarely saw the relevance of ACP to their life, preferring to focus on living well now, that relationships were important in regard to end-of-life decision-making, and that ACP as a concept was often unclear. Enablers for ACP were ensuring frail elders understood what ACP was and could mean for them, treating ACP as an everyday conversation using an honest but frank approach, and engaging frail elders with the process of ACP early. The analysis, synthesis and summation were then discussed and agreed by the research team and a list of potential behaviours to target in the intervention developed.

The list of behaviours was then refined and prioritised. This list included all potential behaviours generated from the synthesised findings of the review, survey, and interviews. As the review and survey had mainly represented H&SCPs views, more weight was given to data generated from the interviews than data generated from the survey. This was to ensure the frail elder and family voice was fully represented and to try to ensure the intervention was grounded in the experiences, attitudes and actualities of frail older people and those closest to them. The potential behaviours were then assessed as to their potential: impact of behaviour change; likelihood of behaviour change; impact on other behaviours; and ease of measurement. Behaviours that scored “very promising” in both impact and likelihood of behaviour change were selected. Prioritisation was iterative and included one author (SC) initially prioritising the list, one author (CJN) reassessing a random selection behaviours, discussion with the research and Advisory teams, and feedback from H&SCPs. Six behaviours were established (Table 3).

**Table 3:** Target behaviours

Overarching behaviour	Frail elders are more likely to engage with ACP if:
Prepare frail elders for ACP conversations	• They understand what ACP means • They know what will be discussed • They are given time to prepare • They are given the opportunity to involve those important to them.
Use the right approach to ACP conversations	• H&SCPs treat ACP as a normal, everyday conversation • H&SCPs use an honest and frank approach that moves at the individual’s pace • H&SCPs use a light-hearted approach, gentle language, and humour, where appropriate.
Make ACP relevant for the older person	• They understand the relevance of ACP to their life. H&SCPs can assist this by: • using frail elder’s past healthcare experiences, or those of family/friends, vignettes, storytelling, or reminiscence • explaining frailty’s likely trajectory to frail elders and their families • explaining key triggers to instigate ACP for frail elders
Remember relationships in ACP	• They are given the opportunity to involve those important to them. • H&SCPs develop rapport and trust.
Lead ACP conversations with living well now	• H&SCPs start conversations by establishing current goals/what matters now to the frail elder • H&SCPs avoid linking ACP solely with planning for the future, dying and death.
Communicate and confirm understanding of ACP with frail elders	• H&SCPs use clear, concise language, explain what ACP is and why it may be relevant to the frail elder • H&SCPs summarise conversations and options, and check understanding.

To begin creating an intervention from these initial six target behaviours, a behavioural diagnosis was conducted using COM-B in conjunction with the Theoretical Domains Framework (TDF) [55, 56]. A behavioural diagnosis shows the behaviour changes that are required. The behavioural diagnosis here established what changes in H&SCPs capability, opportunity and motivation would most likely achieve an increase in frail elders’ engagement with ACP. The TDF is a framework that can be used alongside COM-B to support the identification of what is likely to influence performing a behaviour. As such the TDF can help to understand clinician behaviour and support the implementation of evidence-based practice [56, 57]. In this study the TDF enabled a more contextualised, granular level of analysis, which is particularly useful when diagnosing complex behaviours [37, 39], such as ACP. Behaviours which placed competing demands on H&SCPs, such as documentation and other clinical priorities, were also considered. These were included in the behavioural diagnosis where they were felt to be crucial to professionals increasing frail elders’ ACP engagement. Behaviours were removed if they were outside the scope of the intervention, for example those that required substantial organisational support or a system-wide or cultural change, such as implementing a standardised cross-sector ACP documentation storage and retrieval system, or changing the culture around death and dying. Iterations of the behavioural diagnosis were discussed and agreed with the research and Advisory teams.

Table 4 demonstrates the behavioural diagnosis. It shows the changes required to increase frail elders’ engagement with ACP, the evidence to support this, and is mapped to COM-B and the TDF. This behavioural diagnosis was specified for specialist palliative care H&SCPs, rather than H&SCPs in general. This specialist group were chosen because restrictions during the COVID-19 pandemic meant it was more feasible to bring together H&SCPs based at a hospice than a wider group of community-based staff.

**Table 4:** Changes required to enable specialist palliative care H&SCPs to engage frail elders with ACP

**What needs to happen for the target behaviour to occur?**	**Evidence to support the need for change** *Evidence includes all data sources unless otherwise noted:* *1. Interviews, 2. Integrative review 3. Survey*	**COM-B**^**a**^ **& TDF domain**^**b**^
**Health and social care professionals (H&SCPs) need an awareness of:**		
Why ACP is relevant to frail elders and why ACP needs to start early.	Frailty brings potential for fluctuating capacity & sudden deterioration (2, 3) Prognostication is difficult (2) Physical and psychological capacity likely reduce over time (2) Not engaging in ACP can lead to inappropriate hospitalisations, under- over-treatment, can be burden for family if they do not know what frail elder wanted. (2, 3) Crisis decision-making is difficult (2,3) Time needed to understand relevant concepts	Psych cap/ Know
What ACP means for frail elders	Frail elders/families misunderstanding what ACP is Lack of ACP relevance/importance of living well now for frail elders Focus on shared rather than autonomous decision-making Importance of relationships	
**H&SCPs need to know:**		
The key triggers to instigate ACP for frail elders	Prognostication difficult (2, 3) Triggers often not acted on Can help with relevance for all stakeholders, and decision-making	Psych cap/ Know
Why to use the individual’s previous experiences, scenarios, vignettes, storytelling, reminiscence, to help demonstrate ACP relevance	Previous experiences can encourage engagement and help make ACP more relevant (1, 2) Can encourage engagement, demonstrate and explain ACP’s usefulness, and help make ACP more relevant	
Why to progress ACP conversations at the individual’s pace	Most frail elders happy to engage if conversations go at their pace Going too fast may lead to upset or distress, going too slow may lead to conversations never happening	
Why to correct any misunderstandings regarding ACP	ACP unclear and can be confusing for frail elders and families Lack of shared language can lead to misunderstanding what ACP can offer, what palliative care is, potential of medical treatments etc. Lack of understanding about what ACP is and means can reduce engagement	
Why preparing frail elders for ACP can be beneficial Why including family in preparations can be helpful	Frail elders need time to engage with the concept of ACP Family are important to frail elders, particularly in regards ACP decision-making	
**H&SCPs need the skills to**		
Use language the frail elder/family understand Summarise conversations and confirm understanding Ensure frail elders understand decisions they could, or do, make	ACP language can be confusing or misleading ACP language often vague No shared ACP language Being clear, concise, and checking understanding can help engagement	Psych cap/ Skills
Explain frailty’s likely trajectory to frail elders and their families Explain key triggers to instigate ACP for frail elders	Trajectory is uncertain (2,3) If not understood can mean ACP seems irrelevant to frail elder/family (2, 3) Understanding triggers can help with relevance, for all stakeholders, and decision-making Explaining triggers can help develop relationship with frail elder and family	
Prepare frail elders for ACP conversations Recommend frail elders think about goals and preferences and discusses with family where relevant, prior to ACP Ensure frail elders understand what ACP includes.	Frail elders are more likely to engage in ACP if: They have time to prepare for the conversation They understand what ACP means They know what will be discussed They understand how ACP could be relevant to them Their family are involved to the degree the frail elder wishes.	Psych cap/ Skills
**H&SCPs need to know:**		
Why it’s important to proactively use and create opportunities to engage frail elders with ACP	Many opportunities for ACP are missed e.g. not having the conversation until frail elder in crisis, not starting conversations Proactively creating and using opportunities encourages engagement. Opportunities include triggers, poor prognostic indicators, transitions and cues from frail elders and family.	Psych cap/ Know
Why ACP should be series of conversations rather than a single discussion	Frail elders need time to engage with ACP ACP preferences can change over time Need to build relationships ACP as standard practice would likely increase engagement	
Why ACP conversations should be treated as normal, every-day conversations.	Frail elders prefer a normal, every-day approach to ACP (1)	
**H&SCPs need the skills to:**		
Approach ACP as normal, every-day conversations Use gentle language, a light-hearted approach and, where appropriate, humour Be honest and frank regarding whether current or future care choices are likely/possible e.g. ceilings of treatment, hospice	ACP as standard practice is likely to increase engagement Frail elders recommend using a light-hearted approach, gentle language, and where appropriate, humour (1). Frail elders recommend using an honest and frank approach, that moves at the individual’s pace (1). ACP can be unclear and lead to misunderstandings e.g. what ACP can offer, what palliative care is, potential of medical treatments etc.	Psych cap/ Skills
**H&SCPs need to know:**		
When to encourage family inclusion in ACP Why including/promoting family inclusion is beneficial How to help family understand the frail elder’s wishes and what fulfilling them may mean How to facilitate conversations between frail elders and family	Relationships are important to frail elders Relational decision making is often promoted over individual decision making Family likely to be involved if ACP needs to be enacted, but often do not know the frail elders’ preferences ACP for frail elders is often more about supporting the family	Psych cap/ Know
**H&SCPs need an awareness of**		
Why establishing current goals/what matters now is relevant Why identifying future preferences based on the frail elder’s values, and those they would rather avoid, is relevant How to help frail elders think about parallel planning	ACP as future planning for dying and death is not relevant to many frail elders. Most frail elders focus more on living well now than future planning ACP needs to be relevant to frail elders’ lives for them to wish to engage. Planning for living well now can help frail elders engage with what might happen in the future (parallel planning)	Psych cap/ Know
Explain the relevance of ACP to the individual, their values and beliefs, using clear, understandable terms	ACP as future planning for dying and death is not relevant to many frail elders. Focussing on current values, and those to avoid, can make ACP easier to relate to. Using frail elders past healthcare experiences, or those of family/friends can help engagement	Psych cap/ Skills
**H&SCPs need to:**		
Give frail elders clear, understandable information prior to ACP conversations	Lack of understanding about what ACP means Frail elders need time to engage with the concept of ACP Frail elders are more likely to engage if they know what will be discussed and understand how ACP could be relevant to them	Phy opp/Env
Create a conducive environment to facilitate ACP discussions	ACP more likely to happen in a conducive environment e.g. at home or where the person is comfortable, where there is time, when key people are in attendance (e.g. family), when the conversation is expected.	
**H&SCPs need to see:**		
Other staff facilitating ACP following this approach	Engaged leaders are strong ACP drivers (2) Support/mentoring from colleagues can help overcome ACP barriers and improve skills (2, 3)	Soc opp/ Soc
ACP is relevant for frail elders	Prognostication is difficult Professionals often do not start conversations as they are concerned about upsetting the frail elder or their families	Ref mot/ Bel cons
The benefit of including family in ACP	Relationships are important to frail elders Decisions are often made in relation rather than autonomously	
ACP conversations with frail elders should start early	Frail elders’ uncertain trajectory means they could have a significant deterioration at any time. Starting ACP early allows for frail elders to engage when they have the most physical and psychological capacity Frail elders need time to engage with the concept of ACP and to amend thoughts as things change	
Living well now is relevant to frail elders in regards ACP	Most frail elders prefer to focus on living well now than planning for dying and death. ACP needs to be relevant for frail elders to wish to engage. Planning for living well now can help frail elders engage with what might happen in the future (parallel planning)	
Using a gentle, honest approach will help frail elders engage with ACP	Frail elders recommend using a light-hearted approach, gentle language, honesty, and where appropriate, humour (1).	
**H&SCPs need to:**		
Create reminders to trigger introducing the concept of ACP to frail elders prior to conversations.	Frail elders are more likely to engage in ACP if: They have time to prepare for the conversation They understand what ACP means They know what will be discussed They understand how ACP could be relevant to them	Aut mot/ Reinf
Establish a routine of reassessing ACP decisions every 6 months, or following an ACP trigger	Frail elders’ uncertain trajectory means they could have a significant deterioration at any time. (2,3) Reassessing regularly means the frail elder is given the opportunity to reassess decisions as things change (2,3)	
Create triggers to remember to promote and include family in conversations	Relationships are important to frail elders and can impact ACP decision-making Family likely to be involved if ACP needs to be enacted, but often do not know the frail elders’ preferences	

^a^ COM-B components: *Psych cap* = Psychological capability, *Phy Opp* = Physical opportunity, *Soc Opp* = Social opportunity, *Ref mot* = Reflective motivation, *Aut mot* = Automatic motivation. ^b^ TDF domains: *Bel cons* = Belief about consequences, *Env* = Environment context and resources, *Know* = Knowledge, *Reinf* = Reinforcement, *Skills* = Cognitive and interpersonal skills, *Soc* = Social influences

#### 2. Identify which types of intervention and implementation strategies might be effective

Once the changes most likely to enable specialist palliative care H&SCPs to increase frail elders’ engagement with ACP were understood (Table 4), the types of intervention and implementation strategies most likely to bring about these changes were selected. The BCW offers nine intervention types, for example training and incentivisation, and seven implementation strategies, or policy options, for example legislation and guidelines. Each intervention type and policy option was selected based on those recommended by the COM-B component and TDF domain. Each potential option was then assessed for: affordability; practicability; effectiveness/cost-effectiveness; acceptability; side-effects/safety; and equity (APEASE) [37].

#### 3. Identify intervention content and how to deliver it

Next the intervention was fully specified in terms of content and delivery mechanisms. Intervention content was guided by the Behaviour Change Technique Taxonomy (BCTTv1) [58] which comprises 93 behaviour change techniques (BCT). The BCTs are the proposed active intervention components and include goal setting and problem solving. Appropriate BCTs were identified by one research team member (SC) then discussed and revised with a behaviour change expert (GF). The detailed intervention content was then written and the delivery mechanisms, for example telephone and face-to-face, were chosen. The BCTs, intervention content and mode of delivery were discussed, refined and agreed by the research team. The content of the prototype intervention is described in Table 5.

**Table 5:** CLaD intervention content description mapped to intervention type and behaviour change techniques (BCTs)

CLaD content description	Intervention type	Core BCTs
• Understand frailty and the relevance of ACP • Revise/recap fluctuating physical and mental capacity and sudden deterioration. • Discuss impact of not having an ACP for frail elders and family. • Explain the importance of early engagement, and the impact of leaving ACP too late e.g. missing the greatest opportunity to engage physically and cognitively. • Revise/recap triggers for ACP discussions (e.g. hospitalisation, deterioration, infection, family issues).	Education	5.1, 5.6
Shown film section discussing uncertainty. • Discuss as a group how to communicate uncertainty, fluctuating capacity and sudden deterioration with frail elders and families • Discuss and practice communicating key triggers with frail elders and families	Training	4.1, 6.1, 8.1
**Understanding why ACP is different for frail elders** Show film section discussing challenges of ACP to frail elders. Discuss: • lack of clarity and confusion around ACP for frail elders • relevance of ACP for frail elders • shared rather than autonomous decision-making • importance of family and living well now engaging frail elders with ACP • protecting family and family difficulties in engaging.	Education	4.2, 5.1, 5.6
**Living well now** Show film section on living well now. • Revise challenges around relevance of ACP to frail elders. • Explain importance of living well now, in the moment, for frail elders. • Explain importance of establishing what is important to the frail elders in terms of living well now and thinking about planning for the present and short-term future. • Explain and demonstrate strategies for engagement e.g. focussing on the frail elder’s values, including those they would rather avoid. • Explain/demonstrate strategies to explain the relevance of ACP to frail elders. • Discuss and practice how to help explain the relevance of ACP to frail elders focussing on their values and beliefs, and how to talk about parallel planning.	Education and Training	4.2, 5.1, 6.1, 8.1
**Make ACP more relevant for frail elders** • Explain strategies to help engage frail elders with ACP including making ACP more relevant by using frail elder’s health care experiences, or those of family/friends, vignettes, storytelling, or reminiscence	Education	4.2, 5.1
**Prepare frail elders for ACP conversations** Show film section re being prepared. • Explain why frail elders need to have time to prepare for ACP. • Explain the importance of family in preparing for ACP conversations. • Explain the importance of enabling a conducive environment (usually in own home, with their family/friends around them) • Explain and demonstrate strategies for preparing frail elders for ACP, focussing on goals and preferences, and including family e.g. “Next time would like to talk about…” “You might like to think about” “Why not discuss with….”. • Explain the importance/recommend providing frail elder a Hospice/other ACP leaflet and asking them to look at this/discuss with their family prior to the ACP conversation. • Discuss and practice how to help frail elders to prepare for ACP conversations.	Education, Enablement & Training	1.4, 4.1, 4.2, 5.1, 6.1, 8.1, 12.5
**Remember relationships** Show film section re family. • Explain why family are important to ACP decisions for frail elders. • Encourage frail elders to speak to family about ACP/ACP decisions. • Explain and demonstrate strategies for including family in ACP discussions. Discuss and practice: • how to encourage family inclusion; • how to facilitate conversations between the frail elders and their family • how to help family understand the frail elder’s wishes.	Education	4.1, 4.2, 5.1, 6.1, 8.1
**Use the right approach to ACP conversations** Show film section re approach. Explain: • why conducting ACP as normal, every-day conversations is important. • why using every opportunity to engage frail elders with ACP is important. • why ACP as a process is important. • what is meant by gentle language and a light-hearted approach. • the importance of pacing and likely outcomes of taking ACP too fast or slow. Shown film section re being honest and frank. • Explain that frail elders state they prefer an honest, frank approach e.g. whether any current or future care choices are likely or possible e.g. ceilings of treatment, dying in a hospice. • Discuss and practice approaches to ACP for frail elders. • Refer to toolkit which includes relevant language.	Education & Training	4.1, 4.2, 5.1, 6.1, 7.1, 8.1
**Communicate and confirm understanding of ACP** Show film section regarding ACP confusion for frail elders and families. Explain: • the importance of using clear, understandable language and minimising jargon/euphemisms, e.g. using “your values and preferences for your care” or “care wishes” or other clear terminology rather than “ACP”. • the importance of summarising and confirming understanding with frail elders • the importance of managing expectations e.g. recovery potential, medical outcomes, what services may be available and correcting any misunderstandings.	Education & Training	4.2, 5.1, 4.1, 6.1, 7.1
**Overarching support and revision** Revise and recap: • importance of ACP, family and relationships in decision-making, and living well now. • why starting ACP early is important e.g. to allow for potential deterioration and response shift. • that ACP for frail elders is more about shared than autonomous decision-making. • the suggested approach to ACP with frail elders. • why ACP for frail elders needs to be introduced prior to the ACP conversation • why ACP needs revising regularly • why family can be important in preparation for ACP conversations. • H&SCPs given toolkit to prompt recollection of strategies, language, triggers and the importance of preparation, reassessment and family inclusion. • Discuss previous positive experiences of good ACP conversations with frail elders as a group. • Recommend the approach is thought of as a process, and a strategy to enable frail elders to live their best life, rather than a tick box, one-off exercise. • Advise staff that their approach to ACP for frail elders will be an example to others • Recommend H&SCPs contact the PI or an intervention colleague if they wish to discuss any challenges. • Suggest H&SCPs support each other to develop their ACP skills with frail elders.	Environmental restructuring, Persuasion and Training Modelling and Enablement	4.1, 5.1, 6.1, 7.1, 8.3, 9.1, 12.5,13.1, 13.2, 15.3 3.1, 3.2

Intervention types (from BCW [37]): Education = Increase knowledge/understanding; Enablement = Increase means/reduce barriers to capability/opportunity; Environmental restructuring = Change physical/social setting; Modelling = Provide examples to imitate/aspire to; Persuasion = Provoke positive/negative feelings, motivate action; Training = Develop skills. BCT codes (from BCT taxonomy version 1 [58]): 1.4 = Action planning; 3.1 = Social support (unspecified); 3.2 = Social support (practical); 4.1 = Instruction on how to perform the behaviour; 4.2 = Information about antecedents; 5.1 = Information about health consequences; 5.6 = Information about emotional consequences; 6.1 = Demonstration of the behaviour; 7.1 = Prompts/cues; 8.1 = Behavioural practice/ Rehearsal; 8.3 = Habit formation; 9.1 = Credible source; 12.5 = Adding objects to the Environment; 13.1 = Identification of self as role model; 13.2 = Framing/reframing; 15.3 = Focus on past success

## Results

Following the stages of the BCW, and based on the findings of the integrative review, survey and interviews, the most appropriate intervention types were selected as education and training. The prototype intervention, Conversations on Living and Dying (CLaD) was a 3.5-hours educational skills session for specialist palliative care H&SCPs augmented by a toolkit to support their learning in practice. The lesson plan can be found as Additional file 2. The 3.5-hours included consent, completing a pre-session questionnaire and a 15-min break. During the CLaD session H&SCPs were introduced to contextual factors regarding why ACP is relevant to frail elders and what frail elders think of ACP. Participants were also introduced to strategies for engaging frail elders with ACP. Strategies included starting ACP conversations early, prior to further potential physical or cognitive decline, preparing frail elders for ACP conversations, recognising the importance of relationships and living well now, making ACP relevant for frail elders, using an honest, gentle but frank approach, and ensuring the understanding of all stakeholders. The toolkit (Additional file 3) included the strategies in detail, a shorter checklist, a list of clinical indicators that could be used as triggers for ACP, and vignettes which had been used within the interviews to help participants engage with ACP.

The session was supported by a composite film created from the interviews with frail elders and family members. Interviewees gave consent for either their audio recording (*n* = 2) or filmed interview (*n* = 16) to be included for educational and intervention purposes. This film was used to demonstrate frail elder and family member views on ACP and their suggestions for improving ACP engagement. The session was further supported by participants being given a notebook which they were encouraged to use for reflections during and after the session, and post-it notes, which they were asked to use for questions during the session itself.

Table 5 describes the content of the CLaD educational skills session prototype intervention for specialist palliative H&SCPs, and maps this content to intervention types and BCTs. The prototype intervention was delivered using the lesson plan in Additional file 2. The intervention implementation was guidelines, communication/marketing and service provision. As the intervention was delivered during the first wave of the COVID-19 pandemic, the most feasible and acceptable mode of delivery was a distance educational skills session provided over Zoom. However, the first choice of intervention delivery post-COVID-19 would be face to face with the participants.

### Initial prototype refinement

To refine the CLaD prototype intervention for acceptability, feasibility and potential implementation challenges, it was delivered to 26 specialist palliative care H&SCPs in two group sessions (group 1 *n* = 12, group 2 *n* = 14). Following intervention delivery, participants were asked to trial the prototype in practice before providing feedback. Ethical approval was granted by King’s College London Research Ethics Committee (LRS-19/20–19447, June 2020), and refinement occurred between July and October 2020. Participants were recruited through a large UK urban hospice and gave written consent. Inclusion criteria are reported in Table 1 and participant characteristics in Table 6. Professionals’ experience ranged from very experienced with frail elders and ACP, to never having conducted ACP or rarely working with frail elders. The CLaD prototype intervention was delivered by a member of the research team (SC) over Zoom to the participants who were together in a training room. Participants were supported by two facilitators, both members of hospice staff, and covid-safe protocols were followed throughout.

**Table 6:** Participant characteristics

PROTOTYPE REFINEMENT	Intervention delivery		Feedback	
	*n* = 26		*n* = 24	
	No.	%	No.	%
Nurses	20	76.9	18	75
Occupational therapist	1	3.8	1	4.2
Paramedic	1	3.8	1	4.2
Physiotherapist	3	11.5	3	12.5
Social workers	1	3.8	1	4.2
**Years in practice**			Mean 20.6 (range 3–40 years)	
**Percentage clinical work with frail elders**^a^ (intervention delivery) Mean 60% (range 29–90%)				

^a^ One physio was unable to advise the percentage of time they spent with frail elders

Participants undertook a pre-intervention questionnaire (Additional file 4) focused on their views on ACP and confidence and skills in facilitating ACP with frail elders. Participants then trialled the CLaD prototype for at least one-month in practice with two or more frail elders. Depending on the H&SCPs role and as the trial was conducted during the Covid-19 pandemic, this could be face-to-face in the person’s home, or over the telephone. Professionals then completed a post-intervention questionnaire (Additional file 5). Questionnaire 2 replicated the pre-intervention questionnaire with the addition of questions regarding participants’ views of the prototype and its delivery. Both questionnaires were based on an adapted version of the COM-B behavioural diagnosis. Finally, participants discussed their experience of using the prototype in practice, and ways they would like it to refined (see Additional file 6 for the focus group discussion guide). Due to professional’s availability, they took part in one of six Zoom focus groups (*n* = 23) or an individual Zoom interview (*n* = 1). Further feedback was received from the hospice staff facilitators following each prototype delivery.

Analysis of the feedback showed that overall H&SCPs felt the intervention helped them think differently about ACP for frail elders and encouraged them to engage frail elders more with ACP. Suggested intervention refinements included changes to the toolkit, the addition of role plays, an aide memoire, a tailored document for frail elders and family, and guidance for telephone ACP conversations.

## Discussion

By following a systematic, evidence-based and theory-driven process, we developed and refined the CLaD prototype intervention to increase H&SCPs engagement of frail elders with ACP. The main barriers and enablers to ACP with frail elders were that frail elders rarely saw ACP as relevant to their lives, preferring to focus on living well now, that relationships were important in regard to end-of-life decision-making, and that the concept of ACP was often unclear. These findings are consistent with studies which suggest living in the moment is a coping strategy used by many who are nearing the end of life [59, 60], and that future planning is particularly challenging for those living with daily uncertainty [61], such as frail elders. Similarly, our finding that frail elders often prioritised decision-making within relationships over personal, autonomous decision-making, is consistent with studies looking at autonomy in older people [62–64]. Enablers to ACP included early engagement, using an everyday approach and making ACP clearer for frail elders and families. These findings agree with literature suggesting that early engagement not only provides frail elders the greatest capacity to engage physically and intellectually with the concept of ACP [16, 18], but also conceptualises ACP as a process and part of usual care [65–67].

Previous interventions targeted at improving ACP for frail elders have focused on place of care [22, 23], or advance directive completion [24–29], and although ACP is a complex behaviour, few have been underpinned by behaviour change theories. The PREPARE intervention [68–70] is underpinned by an amalgam of behaviour change theories. A recent feasibility trial including the website and a toolkit for case managers was conducted with 12 frail adults (≥55 years old) and 9 case managers [71]. The authors reported that at 1 week, their toolkit increased case managers’ confidence, attitudes, and readiness to facilitate ACP, and frail elders’ readiness to engage. The most widely used behaviour change theory in ACP appears to be the transtheoretical model of behaviour change, which has been used to develop personalised ACP promotion materials for relatively well (7% self-reported fair to poor health) older adults [72]. However, the model has mainly been used to explore ACP in general, for example, understanding patients’ perspectives and experiences [73], ACP constructs and decision-making [74, 75], or to review interventions for cancer patients [76].

The BCW, COM-B and the TDF have between them been used to develop multiple interventions relevant to older people including audiology [42, 77], pressure care [78], stroke [79], rheumatoid arthritis [80], lower back pain [81], and multimorbidity medication management [82]. In regards to ACP, COM-B and the TDF have been used to develop community-based intervention strategies to support ACP education and participation [83]. However, only 34% of the community groups included represented older people. This study is therefore the first to use the structured approach of the BCW to develop an ACP intervention for frail elders.

Use of the BCW enabled the intervention to be informed by current evidence and multiple stakeholder views, including extensive PPI and Advisory group input. Further, the BCW enables the consideration of context, systems, and working mechanisms, and as such addresses many of the critiques often levelled against ACP intervention development [30, 36]. Using the BCW and TDF, we established key domains that required behaviour change, principally Knowledge and Skills, and the types of intervention most likely to bring about that change, primarily Education and Training. The resultant mapping to BCTs led to an evidence-based, targeted prototype intervention, whose working mechanisms, the BCTs, can be replicated and used in future feasibility testing [37]. The study was further strengthened by initial prototype refinement with H&SCPs who reported that the intervention enabled them think differently about ACP for frail elders and encouraged greater frail elder engagement.

System-wide behaviour change is required [18] to implement ACP with frail elders. This includes behaviour change for frail elders, family, and organisations as well as H&SCPs. Limitations of this study include the inability to address all barriers and enablers to ACP for frail elders. Potential frail elder and family barriers, such as understanding the language of ACP, were categorised as behavioural antecedents and were addressed as contextualisation within the intervention. Further studies are needed to explore significant organisational or system barriers as these were outside the scope of this research. The findings of this study suggest key organisational enablers might include linking policy to funding, accreditation and financial incentives. Further system-wide enablers included developing community-wide support and education programmes to raise ACP awareness and reduce cultural stigma, for example through media campaigns and local volunteer information stands.

A further limitation was that the prototype was initially intended for community-based, generalist H&SCPs. However, as previously discussed, restrictions during the COVID-19 pandemic meant it was more feasible to bring together H&SCPs based at a hospice than a wider group of community-based staff. The intervention was therefore designed for, and refined with, specialist palliative care H&SCPs, although not all of these participants undertook ACP, or worked with frail elders, as part of their regular practice. However, one of the strengths of the BCW is its systematic, structured approach, and therefore, the behavioural diagnosis (Step 1. Understand the behaviour) can be revisited and specified for a broader group of H&SCPs, and the intervention tailored according to their specific behavioural needs. This revisiting and re-specification will take place prior to future feasibility testing, and could be used to revise, specify and tailor the intervention for H&SCPs in other countries and settings. The initial prototype refinement exercise suggested ways the intervention could be strengthened. These suggestions will be considered for the next iteration of the intervention.

## Conclusions

The BCW was used to develop CLaD, an educational skills session for H&SCPs augmented by a toolkit. The CLaD intervention is evidence-based, theory-driven and person-centred, and seeks to increase H&SCPs engagement of frail elders in ACP discussions. Further refinements and feasibility testing now needs to be conducted. However, findings from the initial prototype refinement found the prototype intervention to be acceptable, feasible, engaging, and valuable in clinical practice.

## Supplementary Information

**Additional file 1.** Guidance for the reporting of intervention development studies in health research (GUIDED) checklist, including associated Template for Intervention Description and Replication (TIDieR) checklist.

**Additional file 2.** Lesson plan for the delivery of the CLaD prototype intervention.

**Additional file 3.** The CLaD prototype toolkit consisting of the clinical strategies in detail, a shorter strategies checklist, a list of clinical indicators to be used as ACP triggers, and vignettes.

**Additional file 4.** Pre-intervention questionnaire.

**Additional file 5.** Post-intervention questionnaire.

**Additional file 6.** Focus group discussion guide.

## Data Availability

The datasets used and/or analysed during the current study are included within the article, and its supplementary files, and/or are available from the corresponding author on reasonable request.

## Abbreviations

ACP: Advance care planning
APEASE: Affordability, practicability, effectiveness and cost-effectiveness, acceptability, side-effects and safety, equity
BCT: Behaviour change technique
BCTTv1: Behaviour Change Technique Taxonomy
BCW: Behaviour Change Wheel
COM-B: Capability, opportunity, motivation, behaviour
Frail elder/Frail elders: Cognitively able, domestic-dwelling older person/people living with frailty
GUIDED: Guidance for the reporting of intervention development studies in health research
H&SCPs: Multidisciplinary health and social care professionals
MRC: Medical Research Council Framework for developing and testing complex interventions
PPI: Patient and public involvement
TDF: Theoretical domains framework
UK: United Kingdom
