# Supplementary material for: Development of a theory-based intervention to increase cognitively able frail elders’ engagement with advance care planning using the behaviour change wheel
Source: BMC Health Serv Res. 2021 Jul 20;21:712. doi: 10.1186/s12913-021-06548-4 (PMC8290869; doi:10.1186/s12913-021-06548-4)
Supplement: Supplementary file 1 — Additional file 1. Guidance for the reporting of intervention development studies in health research (GUIDED) checklist, including associated Template for Intervention Description and Replication (TIDieR) checklist. [file 12913_2021_6548_MOESM1_ESM.docx]

#### Additional file 1: GUIDED Template and TIDieR checklist

| Item description | **Area located** | **Page located** |
| --- | --- | --- |
| 1. Report the context for which the intervention was developed. | Background & methods | P 4-13 |
| 2. Report the purpose of the intervention development process. | Background & methods | P 4-13 |
| 3. Report the target population for the intervention development process. | Background & methods | P 4-13 |
| 4. Report how any published intervention development approach contributed to the development process | Background & methods | P 4-13 |
| 5. Report how evidence from different sources informed the intervention development process. | Background & methods | P 4-13 |
| 6. Report how/if published theory informed the intervention development process. | Background & methods | P 4-13 |
| 7. Report any use of components from an existing intervention in the current intervention development process. | N/A |  |
| 8. Report any guiding principles, people or factors that were prioritised when making decisions during the intervention development process. | Methods | P 6-13 |
| 9. Report how stakeholders contributed to the intervention development process. | Background, Methods and Results | P 4-17 |
| 10. Report how the intervention changed in content and format from the start of the intervention development process. | Methods | P 6-13 |
| 11. Report any changes to interventions required or likely to be required for subgroups. | N/a as prototype refinement |  |
| 12. Report important uncertainties at the end of the intervention development process. | Results | P 13-17 |
| 13. Follow TIDieR guidance when describing the developed intervention. | See below |  |
| 14. Report the intervention development process in an open access format. | N/a |  |

**
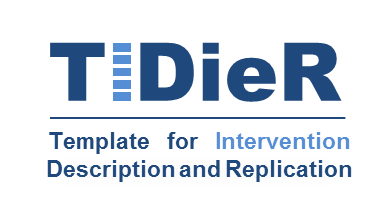
The TIDieR (Template for Intervention Description and Replication) Checklist*:**

Information to include when describing an intervention and the location of the information

| **Item number** | **Item** | **Where located **** | |
| --- | --- | --- | --- |
|  |  | Primary paper  (page or appendix no) | Other ^†^ (details) |
|  | BRIEF NAME |  |  |
| **1.** | Provide the name or a phrase that describes the intervention. | 2-3, & 13-17. | Abstract & Results |
|  | **WHY** |  |  |
| **2.** | Describe any rationale, theory, or goal of the elements essential to the intervention. | 6-17 | Methods & Results |
|  | **WHAT** |  |  |
| **3.** | Materials: Describe any physical or informational materials used in the intervention, including those provided to participants or used in intervention delivery or in training of intervention providers. Provide information on where the materials can be accessed (e.g. online appendix, URL). | 13-17 | Results |
| **4.** | Procedures: Describe each of the procedures, activities, and/or processes used in the intervention, including any enabling or support activities. | 13-17 | Results |
|  | **WHO PROVIDED** |  |  |
| **5.** | For each category of intervention provider (e.g. psychologist, nursing assistant), describe their expertise, background and any specific training given. | 13-17 | Results |
|  | **HOW** |  |  |
| **6.** | Describe the modes of delivery (e.g. face-to-face or by some other mechanism, such as internet or telephone) of the intervention and whether it was provided individually or in a group. | 13-17 | Results |
|  | **WHERE** |  |  |
| **7.** | Describe the type(s) of location(s) where the intervention occurred, including any necessary infrastructure or relevant features. | 13-17 | Results |
|  | **WHEN and HOW MUCH** |  |  |
| **8.** | Describe the number of times the intervention was delivered and over what period of time including the number of sessions, their schedule, and their duration, intensity or dose. | 13-17 | Results |
|  | **TAILORING** |  |  |
| **9.** | If the intervention was planned to be personalised, titrated or adapted, then describe what, why, when, and how. | N/a as prototype refinement | ________ |
|  | **MODIFICATIONS** |  |  |
| **10.^ǂ^** | If the intervention was modified during the course of the study, describe the changes (what, why, when, and how). | 6-17 | Methods & Results |
|  | **HOW WELL** |  |  |
| **11.** | Planned: If intervention adherence or fidelity was assessed, describe how and by whom, and if any strategies were used to maintain or improve fidelity, describe them. | N/a as prototype refinement | ________ |
| **12.^ǂ^** | Actual: If intervention adherence or fidelity was assessed, describe the extent to which the intervention was delivered as planned. | N/a as prototype refinement | ________ |
