# Supplementary material for: Development of a theory-based intervention to increase cognitively able frail elders’ engagement with advance care planning using the behaviour change wheel
Source: BMC Health Serv Res. 2021 Jul 20;21:712. doi: 10.1186/s12913-021-06548-4 (PMC8290869; doi:10.1186/s12913-021-06548-4)
Supplement: Supplementary file 2 — Additional file 2. Lesson plan for the delivery of the CLaD prototype intervention. [file 12913_2021_6548_MOESM2_ESM.pdf]

Additional file 2: Lesson plan

Lesson plan: The CLaD intervention				
	**Session Title**	**Outline**	**Resources**	**Facilitators:**
**09.30**	Introduction	Introduce self, brief re session, consent and questionnaire	Slide 1 – 3, Consent form, PIS, Questionnaire 1, Notepad, Pen, Sweets	Check consent & questionnaires. Collect both.
**09.40**	How the session will run	Explain re Q&A and discussions, set ground rules	Slide 4	
**09.45**	Why we need to look at ACP differently for frail elders	Why is recognising frailty important for ACP? Revise frailty trajectory. Discuss reasons frail elders don't engage with ACP and what this means.	Slides 5-8	Help facilitate brief discussion
**10.00**	Stretch break		Slide 9	
**10.05**	Explaining the section	Brief overview of research project<br>Outline 3 challenges & how section will run.	Slides 10 – 12	
**10.10**	How ACP is experienced by frail elders & families; & Strategies for engaging frail elders with ACP.<br>Part 1: ACP is unclear	Present and discuss challenge. Present and discuss strategies: *Clarity and understanding; Use an honest, frank approach; Being prepared*.<br>Participants discuss and practice how they might use strategies. Q&A.	Slides 13 – 23<br>Film 1 – 06.00<br>Film 7 – 01.20<br>Pink & blue ACP booklets	Help facilitate discussion & Q&A. Flag to pick and blue ACP leaflets.
**11.00**	Tea break		Slide 24	
**11.15**	Part 2: It's more about family and relationships than autonomy	Present and discuss challenge. Present and discuss strategy: *Remember relationships*.<br>Participants discuss and practice how they might use strategy, particularly: Encourage family inclusion; Help facilitate ACP discussions between frail elder & family; Help family understand frail elder's wishes, and what they might mean for them. Q&A.	Slides 25 – 29<br>Film 2 – 05.14	Help facilitate discussion & Q&A
**11.55**	Part 3: ACP is not relevant	Present and discuss challenges. Present and discuss strategies: *Making ACP relevant; Leading with living well now*; Stretch break; *Use an honest, gentle but frank approach.* Remind that individuality is key.<br>Participants discuss and practice how they might use strategies. Q&A.	Slides – 30 - 46<br>Film 3 – 03.11<br>Film 4 – 01.50<br>Film 5 – 02.37<br>Film 6 – 02.10	Help facilitate discussion & Q&A
**12.40**	Summary & bringing it all together	Review session plan, revise challenges and strategies, revise toolkit.	Slide 47 - 50	
**12.50**	Next steps	Remind re next research steps. Adv re support (lecturer & colleagues). Q&A. Thankyou. Credits. Contacts.	Slide 51 - 56	Help facilitate Q&A
**13.00**	Finish			
