# Supplementary material for: Development of a theory-based intervention to increase cognitively able frail elders’ engagement with advance care planning using the behaviour change wheel
Source: BMC Health Serv Res. 2021 Jul 20;21:712. doi: 10.1186/s12913-021-06548-4 (PMC8290869; doi:10.1186/s12913-021-06548-4)
Supplement: Supplementary file 3 — Additional file 3. The CLaD prototype toolkit consisting of the clinical strategies in detail, a shorter strategies checklist, a list of clinical indicators to be used as ACP triggers, and vignettes. [file 12913_2021_6548_MOESM3_ESM.pdf]

FUNDED BY NIHR | National Institute for Health Research

St Christopher's More than just a hospice

King's College London

Helping engage older people living with frailty with advance care planning:

Strategies

Older people living with frailty are extremely vulnerable to sudden health deteriorations and have a reduced ability to recover. This makes helping older people with frailty to engage with ACP a priority. The eight strategies below appear to be important in engaging older people living with frailty with ACP. Suggested phrases should be adapted to the person and context.

**Start early**	This gives the older person living with frailty the greatest chance to engage physically and cognitively, at their own pace, and to make and revise decisions over time.<br><br>Starting early can also help in developing a trusting relationship between you, the older person and their family. Trusting relationships are important for older people with frailty when they think about engaging with ACP.<br><br>Starting early also provides the opportunity to promote ACP as an ongoing conversation. ACP for older people with frailty is rarely a one-off, tick-box exercise.
**Prepare**	Older people living with frailty appreciate time to prepare for conversations regarding their future. This time offers them the opportunity to think about their values and preferences, and to discuss these thoughts with family and those important to them.<br><br>Tell the older person that you would like to begin thinking about their future care preferences in your next conversation. Provide them with any useful, brief ACP resources. Ask them to begin thinking about their goals and preferences for their future care and any questions they have for you.
**Remember relationships**	Relationships are important for older people living with frailty, particularly when it comes to making ACP decisions. Many older people prefer to make shared-decisions, or wish for family or professionals to make ACP decisions for them, although they often do not tell their family what their preferences are.<br><br>Encourage family involvement as desired by the older person. Suggest they speak with their family/those important to them about their wishes before they speak with you. Suggest a family member attends the ACP conversation. Ask the older person if they have a surrogate decision maker or would like help deciding on one. You may also need to help the older person have these conversations with their family.<br><br>If possible, you should conduct ACP conversations with older people you already have a trusting relationship with. This is not always possible, so rapport and trust need to be developed swiftly. If you do not know the person, it may be useful to also discuss ACP with their wider care team prior to any ACP conversations.
**Lead with living well now**	Older people living with frailty are often more interested in living well day to day than planning for the future. It can be useful to begin the ACP conversation focussing on goals and preferences that are important for living well in the moment. This can also help illustrate their personal values.<br><br>Ask them *"What is important to you now?"* This can be used as both a preparation question and within ACP conversations. Discuss what they value as promoting their quality of life, and what they would prefer to avoid.<br><br>Parallel planning may also be useful. After asking "*What is important to you now?*" or a similar phrase, ask them about planning for the worst while hoping for the best, for example, *"If you were to become ill, for example (relevant illness) what would you like to happen?"*

©King's College London

**Make ACP relevant**	Many older people living with frailty do not believe ACP is relevant to them. This is often related to the unpredictability of the frailty trajectory.<br><br>Be frank and explain honestly, but gently, why ACP may be relevant to the older person. This may include discussing their likely trajectory, the impact of contracting a significant illness, the realistic outcome of medical treatments, or managing expectations.<br><br>Discussing the older person's personal experiences (e.g. hospitalisations, infections) may help them recognise the potential relevance of future planning. Vignettes, storytelling, reminiscence or the paper boat analogy may also help someone begin to engage.
**Use an honest, gentle, but frank approach**	Older people living with frailty want ACP to be treated as normal, everyday conversations. This includes being mindful of and using opportunities provided by the older person or their family, for example if they raise subjects such as their last hospital admission or how they feel less well.<br><br>This approach also includes being honest but gentle with your language, using direct but open questions, for example *"If you were to become seriously unwell due to an illness such as (relevant to them), how would you like to be cared for?"* or *"What is your understanding of your (heart disease/diabetes etc)?"* Do not call older people living with frailty 'frail', they are unlikely to recognise themselves as frail and may find it offensive.<br><br>Be aware of matching the older person's pace. They may require time to think and discuss with family before they make decisions.
**Clarity and understanding**	ACP can be confusing for older people living with frailty and their families.<br><br>Use clear language and try to avoid jargon or euphemisms, these can lead to confusion, upset and potentially longer or repeat conversations. ACP as a phrase is a case in point. Try and avoid using "ACP" with older people living with frailty as in most cases it is either confusing or heard as just meaning preferred place of care, death and CPR decisions. Future planning for older people living with frailty is far more holistic, comprising of current and future wishes. Try using "*your values and preferences for your care*", "*care wishes*" or "*future planning*" instead.<br><br>When discussing medical terms, try to discuss "treatment options" rather than "ceilings of care". Be explicit about potential treatment options, for example fluids, antibiotics or oxygen, as relevant to ensure understanding. When discussing CPR, use the phrase "Do not attempt cardiopulmonary resuscitation" rather than "Do not resuscitate/attempt resuscitation", and be explicit as to what CPR as an intervention means.<br><br>Check back to ensure their understanding and also summarise the main points to check your understanding.
**And plan**	Using the above strategies, ensure the older person living with frailty has everything they need to make informed decisions. Correct any misconceptions, explain any confusions, and if family were not involved in the conversation, ask how you can support the older person to discuss their ACP decisions with them. Discuss surrogate decision makers and Lasting Power of Attorney. Document accordingly.

But remember: You may use all the strategies above and the older person may simply not be interested in planning for their future for a multitude of reasons. If this is the case, recommend they may wish to think about a surrogate decision maker and/or appointing a Lasting Power of Attorney, and document accordingly.

This document presents independent research funded by the National Institute for Health Research (NIHR). The views expressed are those of the author(s) and not necessarily those of the NHS, the NIHR or the Department of Health and Social Care.

Helping engage older people living with frailty with advance care planning:

Checklist and Resources

This checklist has been developed for experienced health and social care professionals who have attended the session regarding ACP for older people living with frailty, are already knowledgeable about the process of ACP, and who are generally confident in facilitating ACP in practice.

This guide offers a checklist as a reminder of the learning at the session, along with some suggested phrases and language to use or avoid that appear to be important in helping specifically to engage older people living with frailty with ACP. Suggested phrases should be adapted to the person and context.

**Start early**	***Introduce the concept of ACP as soon as practical***<br><br>Refer to Triggers for starting to think about engaging older people living with frailty with advance care planning.
**Prepare**	***Prepare the person for the conversation***<br><br>For example: "Next time I wonder if we could talk about…"; "For our next conversation I wonder if you could think about…"<br><br>See Additional resources (below) for suggested information for older people and families.
**Remember relationships**	***Promote family/significant other involvement***<br><br>For example: Suggest the older person living with frailty may wish to speak to their family/significant other prior to the ACP meeting. Suggest the older person invites a family member/significant other to the ACP meeting; Help facilitate a discussion between the older person and family member/significant other regarding decisions.
**Lead with living well now**	***Start the conversation with what is important to maintain the person's quality of life now***<br><br>For example: What is important to you now? What helps you live well now? What brings quality to your life? What makes your life meaningful? What do you value most when it comes to maintaining your quality of life? What are your goals?<br><br>***Then move the conversation to the future***<br><br>For example: If you were to become ill, for example (relevant illness), what would you like to happen?... and what would you prefer to avoid? Is there anything you worry about or fear happening? What would you trade for the chance of getting more of what is important to you?<br><br>***If you think they would be ready to hear it, make a recommendation***<br><br>For example: Based on what I've heard/you've said, I'd recommend x, y, and z. What do you think? Does that represent your preferences/Do I have that right?
**Make ACP relevant**	***Make ACP relevant to the individual***<br><br>For example: How are you feeling at the moment? How was your last hospital/illness experience? Talk about their trajectory so far, their experiences, their likely trajectory. Use the paper boat analogy, vignettes, storytelling, or reminiscence work where appropriate. Example vignettes can be found on the Vignettes handout.

**Use an honest, gentle, but frank approach**	***Be honest and frank, but gentle***<br>For example: What have you been thinking about your health? What is your understanding of your (heart disease/diabetes etc)? If you were to become seriously unwell due to an illness such as (relevant to them), how would you like to be cared for/what would you like to happen?
**Clarity and understanding**	***Use clear language and summarise to ensure understanding***<br>For example:<br>• Rather than "advance care planning/ACP" try *future planning*, *thinking about your values and preferences for your care*, or *care wishes*.<br>• Rather than "ceilings of care" discuss "treatment options".<br>• Rather than "Do not resuscitate/attempt resuscitation" use "Do not attempt cardiopulmonary resuscitation", and explain what CPR means.<br>• Do not call older people living with frailty 'frail', they are unlikely to recognise themselves as frail and may find it offensive.
**And plan**	***Document any decisions, including surrogate decision makers***<br>For example: If you were not able to make decisions for yourself in the future, who would you like to speak for you? Who would you like to help us make decisions if you weren't able to?

Additional resources for helping prepare the older person living with frailty and their family

A leaflet to prepare older people living with frailty for ACP is being developed as part of this study but is not yet available. The following documents and links, while not specifically developed for older people living with frailty, may still be useful to help them prepare for ACP conversations:

Age UK have multiple online resources for older people as follows:

- Talking about death and dying with family: https://www.ageuk.org.uk/globalassets/age-uk/documents/booklets/talking_about_death_booklet_final_version.pdf

- Talking about end of life including with friends and family and their doctor, planning future care, and where they can be looked after at end of life https://www.ageuk.org.uk/information-advice/health-wellbeing/relationships-family/end-of-life-issues/

- Power of attorney, Living will, Writing wills, Financial support, and for the family, details of when a person dies and bereavement support https://www.ageuk.org.uk/information-advice/money-legal/end-of-life-planning/

St Christopher's have two general ACP documents:

- Advance care planning guide (2019) https://www.stchristophers.org.uk/leaflets/advance-care-planning
- Advance care planning: Personal preferences and choices for end of life care (2017) https://www.stchristophers.org.uk/resource/advance-care-plan/

The National Council for Palliative Care has a booklet for general ACP

- Planning for your future care: A guide (2009) https://www.nhs.uk/livewell/endoflifecare/documents/planning_your_future_care[1].pdf

This document presents independent research funded by the National Institute for Health Research (NIHR). The views expressed are those of the author(s) and not necessarily those of the NHS, the NIHR or the Department of Health and Social Care.

Helping engage older people living with frailty with advance care planning:

Triggers

Older people living with frailty are extremely vulnerable to sudden health deteriorations and have a reduced ability to recover. Helping older people living with frailty to start thinking about ACP early is therefore a priority. Early engagement provides the greatest opportunity for the older person to engage physically and cognitively, at their own pace, and to make and revise decisions over time.

The following are clinical indicators that suggested someone is coming towards the end of their life. These clinical indicators can act as triggers to engage older people living with frailty with ACP if they have not already engaged. They can also be used to trigger reviews of previously made ACP decisions to discuss if they are still relevant to the older person.

Clinical indicators suggesting possible end of life phase for older people living with frailty

- Diagnosis of moderate to severe frailty (Clinical frailty scale 6 or 7)
- Two or more unplanned hospital admissions in the past 6-12 months or, if 85 or over, one hospital admission
- Persistent and recurrent infections
- Weight loss of 5-10% in the past 6 months
- Multiple morbidity in addition to frailty
- Combined frailty and dementia
- Delirium
- Exacerbation of falling
- Rapidly rising frailty score
- Escalating patient, family or service provider distress
- Older person asking for palliative care support and/or withdrawal of current treatment

Nicholson (2020) Identification of end of life https://www.bgs.org.uk/resources/end-of-life-care-in-frailty-identification-and-prognostication

This document presents independent research funded by the National Institute for Health Research (NIHR). The views expressed are those of the author(s) and not necessarily those of the NHS, the NIHR or the Department of Health and Social Care.

Helping engage older people living with frailty with advance care planning:

Vignette examples

These vignettes were used in the interviews to help the participants, older people living with frailty and their families, engage with the concept of ACP. They are provided here as examples of how you may wish to try and help make ACP relevant for older people living with frailty where required.

Vignette 1: Florence

Florence wanted to remain at home for her care until the end of her life, being supported by her family and the professionals caring for her. But, if her health worsened significantly, she wanted to move to a nursing home. She didn't want to receive cardiopulmonary resuscitation. If she ever needed medical treatment, such as for an infection, she only wanted treatments that could be given in her own home even if that meant it would shorten her life. She had discussed her wishes with her son, who was her main carer, and she had written an advance care plan to let people know her wishes. This was held by her and her GP. Day to day the most important thing for Florence was to support her family and help them cope with life when she had gone, this included writing a will.

Vignette 2: Fred

Fred had been in hospital several times over the last few months. While he would rather not go back again, both Fred and his wife felt it was important for him to be given every opportunity to live for as long as possible. He wanted to receive full, active treatment as needed, including going into intensive care and having a feeding tube if he could not take food by mouth etc. He also wanted to receive cardiopulmonary resuscitation, although he understood this would be a decision made by the clinical team at the time, and may not restore him to good health. For Fred, while he would rather have both quality and quantity of life, quantity was the most important.

Vignette 3: Marjorie

Marjorie didn't want to specify an advance care plan around her future care wishes. She believed what would be would be, trusting her family to make the right decisions if she was ever in a position not to be able to make her own decisions in the future. For Marjorie the important thing was living each day to the full and not wasting the time she had. She focused on "day to day" planning for doing the things she wanted to do, such as deadheading the roses, having a meal out with her family, and carrying out the exercises she had been given to help keep her on her feet. She felt this "day to day" planning, which allowed for how she was feeling each day, brought some order to her life.

This document presents independent research funded by the National Institute for Health Research (NIHR). The views expressed are those of the author(s) and not necessarily those of the NHS, the NIHR or the Department of Health and Social Care.

 ©King's College London
