# Supplementary material for: Development of a theory-based intervention to increase cognitively able frail elders’ engagement with advance care planning using the behaviour change wheel
Source: BMC Health Serv Res. 2021 Jul 20;21:712. doi: 10.1186/s12913-021-06548-4 (PMC8290869; doi:10.1186/s12913-021-06548-4)
Supplement: Supplementary file 4 — Additional file 4. Pre-intervention questionnaire. [file 12913_2021_6548_MOESM4_ESM.pdf]

Questionnaire 1

This questionnaire relates specifically to your experiences when facilitating advance care planning (ACP) with people aged 65 and over, who:

- Are living with frailty (as defined by the clinical frailty scale); and
- Live in the community in their own home; and
- Have capacity to take part in ACP conversations.

About you

Please complete the following questions so that we understand more about you and your practice:

1. Profession	
2. How many years have you been in practice?	
3. What percentage of your current workload is spent working with frail elders?	

Your views on ACP

Think about when you facilitate ACP with frail elders.

4a. Which of the elements below do you usually cover? Please tick all that apply.

4b. Which of these elements would usually be your three top priorities? Please circle the relevant three ticks.

**Element**	**Elements I usually cover**
Ceilings of treatment (highest level of intervention deemed appropriate)	
Current care goals	
Discussing future care needs the frail elder may have	
Discussing likely trajectories and what this might mean for the frail elder	
Discussing medical treatments the frail elder may require	
Discussing social needs the frail elder may have	
Discussing medical investigations and interventions the frail elder does not want	
Do not attempt cardiopulmonary resuscitation	
Establishing legal forms of ACP while the frail elder has capacity	
Preferred place of care	
Preferred place of death	
Who the frail elder would like to be involved in ACP conversations now	
Who the frail elder would like to be involved in decision-making if they lose capacity in the future	
Other	

4c. If you answered ‘Other’ please specify here:

Your confidence and skills in helping engage frail elders with ACP

5. Consider each statement below and tick to show how much you agree or disagree with its sentiment.

**Statement**	**Agree strongly**	**Mostly agree**	**Mostly disagree**	**Disagree strongly**
ACP is not part of my clinical role.				
I am confident answering questions related to purpose and meaning of life when a person is nearing death.				
I am confident when facilitating ACP with frail elders.				
I am confident managing cultural and/or family sensitivities.				
I am not sure when to start facilitating ACP conversations with frail elders.				

6. If you would like to say more about any of the confidence and skills questions, please do so here:

7. If there are other things that impact your confidence or skills in facilitating ACP for frail elders that we have not mentioned, please list them here:
