# Supplementary material for: Development of a theory-based intervention to increase cognitively able frail elders’ engagement with advance care planning using the behaviour change wheel
Source: BMC Health Serv Res. 2021 Jul 20;21:712. doi: 10.1186/s12913-021-06548-4 (PMC8290869; doi:10.1186/s12913-021-06548-4)
Supplement: Supplementary file 5 — Additional file 5. Post-intervention questionnaire. [file 12913_2021_6548_MOESM5_ESM.pdf]

FUNDED BY NIHR | National Institute for Health Research

KING'S College LONDON

Questionnaire 2

This questionnaire relates specifically to your experiences when facilitating advance care planning (ACP) with people aged 65 and over, who:

- Are living with frailty (as defined by the clinical frailty scale); and
- Live in the community in their own home; and
- Have capacity to take part in ACP conversations.

About you

Please complete the following questions so that we understand more about you and your practice:

1. Profession	
2. How many years have you been in practice?	
3. What percentage of your current workload is spent working with frail elders?	

Your views on ACP

Think about when you facilitate ACP with frail elders.

4a. Which of the elements below do you usually cover? Please tick all that apply.

4b. Which of these elements would usually be your three top priorities? Please circle the relevant three ticks.

**Element**	**Elements I usually cover**
Ceilings of treatment (highest level of intervention deemed appropriate)	
Current care goals	
Discussing future care needs the frail elder may have	
Discussing likely trajectories and what this might mean for the frail elder	
Discussing medical treatments the frail elder may require	
Discussing social needs the frail elder may have	
Discussing medical investigations and interventions the frail elder does not want	
Do not attempt cardiopulmonary resuscitation	
Establishing legal forms of ACP while the frail elder has capacity	
Preferred place of care	
Preferred place of death	
Who the frail elder would like to be involved in ACP conversations now	
Who the frail elder would like to be involved in decision-making if they lose capacity in the future	
Other	

4c. If you answered ‘Other’ please specify here:

Your confidence and skills in helping engage frail elders with ACP

5. Consider each statement below and tick to show how much you agree or disagree with its sentiment.

**Statement**	**Agree strongly**	**Mostly agree**	**Mostly disagree**	**Disagree strongly**
ACP is not part of my clinical role.				
I am confident answering questions related to purpose and meaning of life when a person is nearing death.				
I am confident when facilitating ACP with frail elders.				
I am confident managing cultural and/or family sensitivities.				
I am not sure when to start facilitating ACP conversations with frail elders.				

6. If you would like to say more about any of the confidence and skills questions, please do so here:

7. If there are other things that impact your confidence or skills in facilitating ACP for frail elders that we have not mentioned, please list them here:

Your views on the intervention

Wherever 'the intervention' is mentioned below, this refers to the training session, strategies and toolkit.

8. Once you have attended the training session and used the intervention with at least two frail elders, please consider each statement below and tick to show how much you agree or disagree with its sentiment.

**Statement**	**Agree strongly**	**Mostly agree**	**Neither agree nor disagree**	**Mostly disagree**	**Disagree strongly**
I have a better understanding of the relevance of ACP for frail elders					
I have a better understanding of strategies I can use to help engage frail elders with ACP					
I know when to start ACP conversations with frail elders					
I am confident starting ACP conversations with frail elders					
I have the right mental and physical tools to conduct ACP with frail elders					
I have people around me who support me to facilitate ACP with frail elders					
I make opportunities to discuss ACP with frail elders					
I promote family engagement with ACP conversations					
I believe facilitating ACP for frail elders is a priority					
I have developed a better approach to engaging frail elders with ACP					

9. If you disagreed with any statement please say why here:

10. If you would like to say more about any answer, please do so here:

11. What did you find most helpful or useful about the intervention, and why?

12. Which part of the intervention did you find least useful, and why?

13. What were the challenges of trying to implement the intervention in practice?

14. Which strategies and toolkit elements have you found most useful in practice, and why?

15. Has your practice changed as a result of the intervention? If so, how has it changed?

16. Has the experience of using the intervention in practice made you think of anything that needs changing or adding to the intervention? If so, what do you suggest is changed?

The training session itself

17. Thinking about the training session itself, consider each statement below and tick to show how much you agree or disagree with its sentiment.

Statement	Agree strongly	Mostly agree	Mostly disagree	Disagree strongly
The training was the right length to deliver the content				
The training had the right amount of participants to allow for participation				
The design of the training encouraged participation				
The training was at an appropriate level to understand the content				
The training was relevant to improving my knowledge and skills regarding engaging frail elders with ACP				
The training helped me understand how to apply the strategies in practice				
Applying the knowledge in practice helped reinforce what was taught				
The remote delivery did not make a significant difference to my training experience				
The facilitators in the room helped the session run smoothly				
Larger groups would have been more beneficial for the discussion sections (assuming social distancing was not a necessity)				
The lecturer was knowledgeable about the course content				
The lecturer was responsive to questions and other needs				
I would recommend the training to colleagues				

18. If you disagreed with any statement please say why here:

19. If you would like to say more about any answer, please do so here:

20. If you would like to say anything else about the training session, please do so here:
