# Supplementary material for: Development of a theory-based intervention to increase cognitively able frail elders’ engagement with advance care planning using the behaviour change wheel
Source: BMC Health Serv Res. 2021 Jul 20;21:712. doi: 10.1186/s12913-021-06548-4 (PMC8290869; doi:10.1186/s12913-021-06548-4)
Supplement: Supplementary file 6 — Additional file 6. Focus group discussion guide. [file 12913_2021_6548_MOESM6_ESM.pdf]

Conversations on living and dying: facilitating advance care planning for community-dwelling older people living with frailty

Focus Group topic guide

The aim of the focus group is to gain feedback on the participants' experience of using the intervention in practice. This data collection method will completement the questionnaires by allowing for a broader, richer and deeper discussion regarding acceptability.

The following topic guide will be used to guide discussions:

- How did you find the intervention in practice?
- What worked? (were there any facilitators?)
- What did not work as well? (were there any barriers?)
- Did you feel confident using the intervention?
- Did you feel the intervention was effective?
- Do you think anything should be added to, or changed in, the intervention to make it better?
- Do you think anything should be removed from the intervention to make it better?
- Did you find the training appropriate?
- How could the training be improved?
- Is there anything else that you think would help facilitate advance care planning with older people living with frailty in the community?

There may also be a deeper discussion of any of questionnaire 2 responses as relevant
